# Perceptions of Knowledge, Disease Impact and Predictive Genetic Testing in Family Members at Risk to Develop Early-Onset Alzheimer’s Disease (EOAD) and Their Levels of Suicidal Ideation: A Mixed Study

**DOI:** 10.3390/brainsci13030501

**Published:** 2023-03-16

**Authors:** Yesica Arlae Reyes-Domínguez, Luis E. Figuera, Aniel Jessica Leticia Brambila-Tapia

**Affiliations:** 1 Maestría en Psicología de la Salud, Departamento de Psicología Básica, Centro Universitario de Ciencias de la Salud (CUCS), Universidad de Guadalajara, Guadalajara 44340, Jalisco, Mexico; yesica.reyes2393@alumnos.udg.mx; 2 División de Genética, Centro de Investigación Biomédica de Occidente (CIBO), Instituto Mexicano del Seguro Social (IMSS), Guadalajara 44340, Jalisco, Mexico; luis.figuera@academicos.udg.mx; 3 Doctorado en Genética Humana, Centro Universitario de Ciencias de la Salud (CUCS), Universidad de Guadalajara, Guadalajara 44340, Jalisco, Mexico; 4 Departamento de Psicología Básica, Centro Universitario de Ciencias de la Salud (CUCS), Universidad de Guadalajara, Guadalajara 44340, Jalisco, Mexico

**Keywords:** early-onset Alzheimer’s disease, predictive genetic testing, suicidal ideation, genetic counseling, disease impact, perceptions

## Abstract

Early-onset Alzheimer’s disease (EOAD) is an autosomal dominantly inherited disease, in which a founder effect has been described for A431E mutation in the *PSEN1* gene, with most of the affected patients being residents of a small town in the state of Jalisco in Mexico. To date, no studies have been performed in order to know the impact of the disease on this population. Therefore, the objective of this study was to investigate the perceptions in the knowledge, the impact of the disease and the intention to take the predictive genetic testing in the population at genetic risk of Jalisco. For this objective, we performed a mixed study that included a qualitative methodology (semi-structured interviews), and, in addition, we measured suicidal ideation, stress and depression with quantitative instruments in order to compare them with a control group. Of the 28 invited individuals, 9 accepted to participate, from which, 5 (55.56%) participants did not know their genetic risk to develop the disease and 5 (55.56%) would want to take the predictive genetic testing in order to be prepared to face the disease; however, among those who did not want to know, 2 individuals (22.22%) mentioned that they would consider suicide if they were positive for the pathogenic variant. On the impact of the disease, we detected that the adaptation to the familiar’s needs was the most frequent answer, including changes in their lifestyle (being responsible since very young, changes in social life and familiar dynamic), this being their main stressor, followed by changes in plans for the future and contemplating the possibility of being affected. Although no differences in stress and depression between groups were observed, we detected that suicidal ideation was significantly higher in the group of cases. These results highlight the importance to involve all the family in genetic counseling in order to clarify any doubts and also to attend to them psychologically to prevent suicidal ideation and attempts.

## 1. Introduction

Alzheimer’s disease (AD) is the most prevalent progressive neurologic disorder which affects aged people worldwide [1,2]. However, there are other types of dementias that affect young people, these types are included in young-onset dementias (YODs), which have a frequency of 1 in 1000 people [3], and appear under 65 years old. YODs are a group of diseases that include: early-onset Alzheimer’s disease (EOAD), frontotemporal dementia and rare types of dementia [3,4]. Different from the multifactorial Alzheimer’s disease, most YODs are caused by genetic mutations, which are inherited in an autosomal dominant fashion, which represents a higher psychological burden to the children of the affected patients, considering that they are 50% likely to present the disease [5]. When the mutation present in a specific family is detected, it is possible the performance of predictive genetic testing, which can be offered to the relatives at risk to develop the disease in order to know in advance if they will present it [5]. In some of these genetically inherited dementias, the decision to take the test ranges from 5–30% [6]. A recent review of qualitative studies performed in YOD, which included individuals from different countries, mainly the United Kingdom, United States and Norway, showed that these types of dementias have a considerable psychological impact, including impairment in family functioning with a significant and varied emotional affectation [7]. However, none of the studies presented in this report studied the Mexican population at risk to develop EOAD. Early-onset Alzheimer’s disease is an autosomal dominantly inherited form of Alzheimer’s disease (AD) that represents a prevalence of 5.3/100,000 persons at risk (41–60 years) [8] and is caused by mutations in three different genes: presenilin-1 (*PSEN1*), presenilin-2 (*PSEN2*) and amyloid protein precursor (*APP*), with most of the mutations in *PSEN1* [8]. Each of these genes is considered to play a role in the production and clearance of beta amyloid peptides, important constituents of senile plaques in the brain [9]. Among the mutations reported in *PSEN1*, the mutation A431E, has been identified in more than 85 families in Mexico and the United States; this mutation originated from a founder effect in Jalisco, Mexico [10]; and has a mean age onset of symptoms of 40 years, and a heterogeneous clinical phenotype, including high frequencies of spastic paraparesis, language disorders and neuropsychiatric symptoms with an average life expectancy of 10 years since diagnosis, showing a faster decline in functions and more aggressive clinical course when compared with AD [11]. To date, few studies have been conducted in order to explore the perceptions of some aspects of the disease in this population; however, the knowledge, the impact of the disease, as well as the predictive genetic testing, has been unexplored or poorly explored, with reports performed only in the US population, but not in the original Mexican population in Jalisco [10,12]. The study of this particular population, most of which still live in their hometown, is especially needed, considering that their perceptions could differ from people residing in big cities of more developed countries with more access to information and clinical services. 

The objectives of this study are (a) to explore the perceptions in knowledge and disease impact in individuals at risk to harbor the mutation A431E in the *PSEN1* gene (producing EOAD), in order to determine the genetic and psychological needs of this population, including the intention to take predictive genetic testing, and (b) to perform a quantitative study to compare the levels of psychological stress, depression and suicidal ideation between the studied cases and a control group, this in order to probe the hypothesis that the group of individuals at risk to develop EOAD have higher levels of these three variables.

## 2. Subjects and Methods

Relatives of patients diagnosed with the A431E mutation of the *PSEN1* gene in the genetic service of a third-level attention hospital in Guadalajara, Jalisco, who were at risk to harbor the mutation (potential carriers from 18 to 42 years old) and who lived in the state of Jalisco, Mexico, were contacted by phone call, in which they were told about the objective of the study and in case they wanted to participate, it was performed a date for another phone call to perform the interviews and the measurement of the instruments. 

### 2.1. Qualitative Study

In-depth semi-structured interviews were performed addressing the following domains: (a) doubts about the diagnosis and genetic counseling, (b) changes produced or changes expected for the future because of the familiar´s disease, (c) strategies employed for these changes if any, (d) knowledge about the predictive genetic testing, and (e) if they would like to perform or not this predictive genetic testing and why.

For the qualitative analysis, the interviews were audio-recorded and transcribed verbatim; then, the transcripts were coded into themes, subthemes and codes according to the domains explored and the types of answers obtained. A matrix that summarized the main data obtained with each question was constructed and analyzed (by the 3 research team members) using a thematic framework approach [13]. According to this approach, the data analysis was performed by the identification of all the different answers obtained from the participants (subthemes and codes) mentioned for each question (themes). 

### 2.2. Quantitative Study 

In the group of potential carriers and in a control group of general population (obtained with a snowball technique), matched by age and gender with the group of cases, we performed the following psychological measurement: perceived stress, measured with the Cohen perceived stress scale, which consists of 14 questions with 5 answer options: from “never” (0) to “very frequently” (4), range: 0–4 [14], depression measured with the shortened version of the CESD instrument, which consists of 10 questions with 4 answer options: from “none day” to “5–7 days in the week”, range: 0–3 [15] and suicidal ideation measured with the 3 questions included in the CES-D/IS instrument, with 4 answer options: from “none day” to “5–7 days in the week”, range of 0–3 [16].

### 2.3. Statistical Analysis

The scoring procedure was performed by obtaining the average for each instrument in all the individuals studied (the sum of each response obtained by each subject and divided by the number of items included in the instrument). In the data description, we used the mean and standard deviations for quantitative variables and percentages for qualitative ones, for the comparison of the psychological instruments between groups we used the Mann–Whitney U test, considering the sample size of both groups and to compare sociodemographic qualitative variables between groups we used chi-squared and Fisher exact tests. A *p* value < 0.05 was considered statistically significant. Cronbach´s alpha test was performed for the instruments used. All statistical analyses were performed in SPSS v 25.0.

### 2.4. Ethical Compliance

The protocol was approved by the Ethics and Research Committee of the Health Sciences University Center of the University of Guadalajara (CUCS/CINV) with the registration number CI-08420. The study followed the guidelines of the Declaration of Helsinki and all the participants gave their consent to participate in the study. 

## 3. Results

From 28 potential carriers of the mutation, 14 (50.0%) agreed to be contacted to explain the objective of the project, and from these, only 9 (32.14%) potential carriers belonging to 8 different families, agreed and completed their participation in the interview and instruments measurements. The other contacts, although initially agreeing, did not answer (after several attempts) the programmed second phone call. Of the 9 contacted familiars, 5 (55.56%) were women, and the mean ± SD of age was 27.67 ± 6.71 years. Most participants were single: 5 (55.56%); 3 were married (33.33%) and 1 divorced (11.11%); most had children: 5 (55.56%) and the mean ± SD of siblings of the participants was: 4.67 ± 3.35.

### 3.1. Qualitative Results

**Figure 1:**
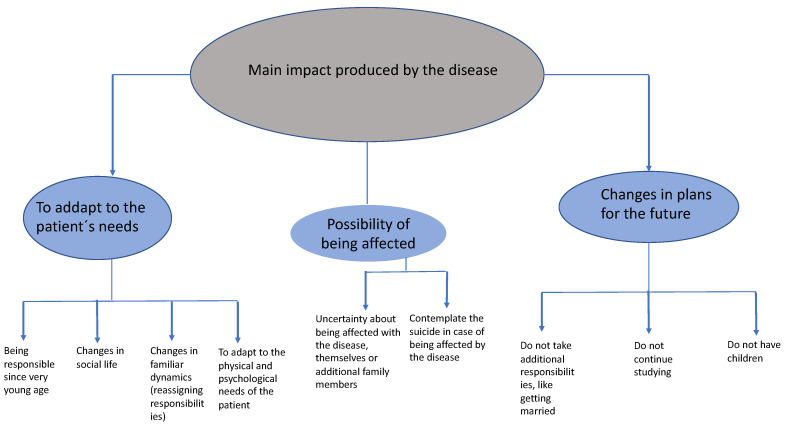
Changes or impacts produced by the disease.

According to the domains explored in the interview, 4 main themes emerged: (a) doubts about the diagnosis and genetic counseling; (b) changes or impact produced by the disease so far or in the future; (c) strategies used to cope with these changes; and (d) knowledge and intention to take the predictive genetic testing (Table 1, Figure 1).

#### 3.1.1. Theme 1: Doubts about the Diagnosis and Genetic Counseling

In this theme, 5 different codes emerged: (1) 5 participants did not know the origin of the disease/how it is produced; (2) 5 participants did not know the likelihood to inherit the disease; (3) 2 participants did not know the average of the time it last/the life expectancy of the patients; (4) 2 participants wanted to know if there is a possible treatment for the disease; and (5) another participant did not know the exact name of the pathology. 

#### 3.1.2. Theme 2: Changes or Impact Produced by the Disease

In this theme 8 subthemes emerged:

(1) Adaptation to the needs of the patient, in this sub-theme the following 5 codes were identified: (a) being responsible younger: 3 participants mentioned have made responsible for the patient since a very young age; (b) changes in the social life (mentioned by 3 participants); (c) changes in the familiar dynamic, reassigning responsibilities (mentioned in 3 participants); (d) adapt to the physical needs of the patients (mentioned by 3 participants): “to be aware that he does not fall, that he takes his medications, be aware of his needs”; and (e) adapt to the psychological changes/needs of the patient (mentioned by 4 participants): “we are all waiting for what emotional state is going to arrive in; because there are times when he arrives crying saying that he no longer wants to live, other times he is very angry, he is very changeable in his moods”.
(2) Changes in plans for the future: this sub-theme had the following codes: (a) do not get married at that moment/do not take additional responsibilities (mentioned by 2 persons), (b) do not continue studying, to be able to care her familiar (mentioned by 2 persons); (c) do not have children or more children (mentioned by 2 participants).
(3) To contemplate the possibility of being affected: to consider that other familiars or themselves be affected by the disease was mentioned by 4 participants.
(4) Regret of having had children, this was mentioned by 2 participants.
(5) Reconceptualize the origin of the disease, 2 participants mentioned that they stopped believing that the disease was a witchraft.
(6) Family disunity: “we are 11 siblings and it seems that we do not even know each other, nobody wants to take responsibility” (mentioned by 2 persons).
(7) Contemplate suicide, 1 person mentioned that he and another familiar at risk considered suicide in case they had the mutation.
(8) To have a healthy lifestyle (mentioned by 1 person).
(9) To live and take advantage of her life: “I want to live my life, to do the things that I have not done, to spend more time with my daughter, to play with her and talk with the older one … I want to live and live” (mentioned by 1 person).

#### 3.1.3. Theme 3: Strategies to Cope with Those Changes

In this theme 5 subthemes emerged:

(1) To develop emotional abilities: this was the main sub-theme mentioned by 6 participants; this included the following 6 codes: (a) empathy, (b) patience, (c) adaptation, (d) acceptation, (e) to speak it, and (f) to be busy in other things.
(2) Search of social support, this sub-theme was mentioned by 5 individuals and it referred to finding a person that helps with the patient´s care: “I think that we are going to choose to hire a person who be in the time that we cannot be with him” (in 4 cases) and to find emotional support in familiars (in 1 case).
(3) Avoid contact with the problem or patient, in order to distract the attention and avoid a bad emotional reaction from the patient (mentioned in 2 cases).
(4) To organize the time and activities (mentioned in 2 cases).
(5) To perform the predictive genetic testing, “to be able to face it from before and do not be thinking all the time if I will also be sick” (mentioned in 1 case).

#### 3.1.4. Theme 4: Knowledge and Intention to Perform the Predictive Genetic Testing

In this theme, we observed the following subthemes: (a) three participants did not know anything about the test, (b) doctor is not prepared: 3 participants referred that the geneticist told them that he was not prepared to perform it because they needed psychological monitoring; and (c) confounded the test: two additional participants confounded the test with other studies that had been performed to them.

When the participants were asked whether they wanted to perform the test or not, 4 persons (44.44%) mentioned that they did not want because of the following reasons: (a) to be afraid: they mentioned being afraid that if they are positive, they think that it would be worse than do not know; even one participant mentioned the possibility of suicide if she is positive: “Imagine, where they tell me that I am positive, I die of depression or even commit suicide to avoid it, I do not want to be a burden for my daughters”, and (b) there is no treatment available (mentioned by 1 participant).

The other 5 participants (55.56%) mentioned that they did want to know for the following reasons: (a) to be prepared: for everything, in legal formalities; (b) to avoid familiar problems: with the romantic partner; (c) to prevent their children because they can also have the mutation and to prevent with a healthy lifestyle, (d) to stop thinking if they have the mutation, (e) to know how much time they have to achieve their goals and (f) in case it is possible, to delay the onset of the disease (each one of these answers was mentioned by 1 participant). 

#### 3.1.5. Results of the Quantitative Study

The Cronbach´s alpha tests showed scores above 0.7 for all the instruments included.

The 9 participants were compared with a control group of 100 individuals of the same state (Jalisco), who were similar in age, sex, schooling and number of children (Table 2). However, these groups were different in the city of origin, with more individuals in the control group originating from the Guadalajara metropolitan area, and in the group of cases, there were more individuals from a place different from Guadalajara metropolitan area. This difference is expected by considering that most affected individuals live in a region located in the north of the state.

In the comparison of the psychological variables: stress and depression, we found that they were similar in the group of cases and the control group, although we observed a slight increase in the levels of stress in the control group when compared with the case group. The suicidal ideation was significantly higher in the group of cases in comparison with the control group (Table 3). In this instrument, we observed that from the 3 questions performed: (a) I had thoughts about death; (b) I felt that my family would be better off if I was dead; and (c) I thought of killing myself; we noticed that the high levels of this variable in the group of cases were due to the fact that all the participants reported the highest frequency in having thought the second phrase: “I felt that my family would be better off if I was death”.

## 4. Discussion

In this study, we explored the perception of the knowledge, disease impact and predictive genetic testing in familiars at risk to develop EOAD in the state of Jalisco, where the A431E mutation originated. With respect to the knowledge, we noticed that although all the patients, and therefore the affected families, were attended by the same geneticist, we observed that not all the participants were aware of the origin of the disease nor its inheritance pattern, with it being ignored by 5/9 (55.56%) participants. These results coincide with the results observed in the familiars of the US population with this mutation, where the authors refer that very few respondents at risk clearly understood their own genetic risk [12]. This emphasizes the need to involve the family in the genetic counseling of the disease and/or perform many talks about the disease to which these familiars are invited. However, different to this previous study, where most participants (72.2%) wanted to perform the genetic testing (predictive genetic testing), in the present study around half of the participants wanted to perform the test, and we believe that this percentage can be overestimated considering the low percentage of participation in the study (32.14%). These differences can be attributed to the population studied, this considering that the US population has more information available by the health system and research teams, so the disease can be normalized and more accepted than in the Mexican population, where most affected families do not live in the Guadalajara metropolitan area, but in towns located in the north of the state. The possible lack of disease acceptance can also be related to the low participation of the familiars invited. This hypothesis coincides with the higher stigma, qualitatively and quantitatively observed in Mexican familiars compared with US familiars of patients with the disease, in studies performed in USA [12]. 

With respect to the reasons to perform the predictive genetic testing, the answers more mentioned were related to being prepared themselves and their families, including the possibility that their children also have the mutation (in one person); however, although 55.56% of participants were interested in performing the predictive genetic testing, only one person mentioned it in the strategies employed to face the changes produced by the disease, either in the present or the future. The other changes mentioned included adapting to the patient’s needs and conditions, which highlights that, in addition to being potential patients, the familiars are also caregivers of the patients. This was more mentioned than the possibility that they are affected or the regret of having children and have transmitted the mutation to their children. These findings are explained by considering that the main stressor in their lives is to be a caregiver and is also explained by the low comprehension of the disease’s nature, including their own risk to present the disease These results coincide with the review performed in qualitative studies in YOD, where the authors report disruption in family functioning as one of the main themes related with the impact of the disease [7]. This disruption is mainly due to the role reversal between affected parents and their children, being the children the caregivers of their parents and assuming more responsibilities at home. Another impact produced by the disease was a change in plans for the future, results that also coincide with the review of qualitative studies performed in YOD [7] where “uncertainty” was a common theme, mentioning that the children of parents with YOD put their lives “on hold” altering or changing future plans, including education, career and family planning. 

In addition, when we analyze the strategies used to cope with the changes produced by the disease, the most mentioned strategies, were the emotional abilities followed by the search for social support and avoidant coping strategies, these answers also are in line with the fact that their main stressor is to be caregivers and not possible patients. These results also coincide with the review reported in YOD [7], where the main coping strategies implemented by children of parents with YOD were physical, cognitive and emotional distancing, along with living the moment, denial and lifestyle changes. 

With respect to the quantitative study, the lack of differences in the levels of depression and stress may be due to the fact that most individuals in the case group live in small towns far from the Guadalajara metropolitan area, while most individuals in the control group originate from the Guadalajara metropolitan area, which is a bigger city that can be more stressful and therefore depressant for its residents. However, despite this difference, it is interesting the high levels of suicidal ideation in the case group, due to the high frequency of the thought that their family would be better off if they were dead, this suggests that they think that can be a burden for their family in the future, which could lead to suicidal attempts. However, it is also interesting that no participant had thought of suicide during the last week, which could be due to the caregiver role that they perform in the family in many cases, which makes them feel needed by their families in the present. These findings coincide with the high levels of suicidal ideation in potential carriers of the mutation in Huntington´s disease [17] with suicidal thoughts and attempts reported being from 3 to 5 times higher than in the general population [17,18]. In addition, suicidal plans and adverse events have been shown to increase in carriers of Huntington´s disease after the predictive genetic testing, in longitudinal studies [17,19]. These findings are also in accordance with the consideration of suicide in two participants if they are positive in the predictive genetic testing, which highlights the importance to address this issue in the health monitoring of these potential carriers.

Some of the limitations of the study are the small sample size, which could diminish the representativeness of the results obtained, either in the qualitative or quantitative study. Another limitation is that there was not possible to include a control group whose birthplace (related to their residence place) was similar to the case group, which could have permitted us to better detect differences in stress, depression and suicidal ideation between groups.

## 5. Conclusions

In conclusion, most familiars at risk to develop EOAD from Jalisco in Mexico were not clearly aware of their genetic risk to develop the disease nor understood the nature of the disease; in addition, their main stressor was to be caregivers of their patients (including changes in family dynamics, role reversal and changes in plans for the future), and in second place to contemplate the possibility to develop the disease and have transmitted it to their children. Only half of the participants wanted to perform the predictive genetic testing, in order to prepare themselves and their familiars; while the other half did not want to perform it, and in two cases contemplated suicide if they were positive, which coincided with the high levels of suicidal ideation in this group when compared with a control group. Therefore, we consider that constant genetic counseling talks should be given in this population, where the possibility of suicide ideation be also attended, in order to inform them about the disease, increase its acceptance and prevent suicides. However, more studies on this population living in the state of Jalisco are needed in order to confirm these results.

## Figures and Tables

**Table 1:** Themes and subthemes of the qualitative study.

Theme	Subthemes
**1. Doubts about the diagnosis and counseling**	(a) Doubts about the origin of the disease (b) Doubts about the likelihood to inherit the disease (c) Doubts about the average time it lasts/the average life of the patients (d) Doubts about possible treatments (e) Doubts about the exact name of the pathology
**2. Changes or impact produced by the disease**	(a) To adapt to the needs of the patients (including changes in lifestyle (social life and familiar dynamic), being responsible from a very young age) (b) Changes in plans for the future (c) Regret of having had children (d) Family disunity (e) Contemplating suicide (f) To have a healthy lifestyle (g) To live and take advantage of life
**3. Strategies to cope with that changes**	(a) To develop emotional abilities (b) Search for social support (c) To avoid contact with the problem or patient (d) To organize the time and activities (e) To perform the predictive genetic testing
**4. Knowledge and intention to perform the predictive genetic testing**	Knowledge: (a) Do not know anything about the test (b) Lack of preparation of the physician´s team in patient´s psychological management (c) Confounded the test with another one Intentions to perform the test Reasons to perform the test: (a) To be prepared if they are positive (b) To avoid familiar problems (c) To prepare their children and themselves with a healthy lifestyle (d) To stop thinking about the possibility of having the mutation (e) To know how much time they have to achieve their goals (f) To delay the appearance of the disease (if possible) Reasons to not perform the test: (a) Being afraid of being positive because there is no treatment, and they could contemplate suicide

**Table 2:** Sociodemographic data of participants included in both groups.

Variable	Cases, n = 9	Control, n = 100	*p* Value
Age, mean ± SD	27.67 ± 6.71	34.19 ± 10.97	0.085
Female, n (%)	6 (66.7)	71 (71.0)	0.712
Schooling, n (%)			0.080
Preschool	0 (0.0)	2 (2.0)	
Elementary school	1 (11.1)	9 (9.0)	
High school	6 (66.7)	18 (18.0)	
Preparatory	2 (22.2)	55 (55.0)	
Bachelor’s degree	0 (0.0)	6 (6.0)	
Technical career	0 (0.0)	9 (9.0)	
Postgrad	0 (0.0)	1 (1.0)	
Number of children, median (range)	1 (0–3)	1 (0–6)	0.491
Birthplace in Guadalajara metropolitan area	3 (33.3)	95 (95.0)	<0.001

*p* values obtained with Mann–Whitney U test for quantitative variables and Fisher exact test for qualitative ones.

**Table 3:** Comparison of stress, depression and suicidal ideation between groups.

Variable, Mean ± SD	Cases, n = 9	Control, n = 100	*p* Value *
Stress	1.07 ± 0.22	1.71 ± 0.63	0.052
Depression	1.06 ± 0.27	1.10 ± 0.64	0.172
Suicidal ideation	1.29 ± 0.53	0.28 ± 0.64	<0.001

* *p* value obtained with Mann–Whitney U test.

## Data Availability

Data that support the information of the study will be available upon a reasonable request.
